# Supplementary material for: Selective Oxidation of Transient Organic Radicals in the Presence of Gold Nanoparticles
Source: Nanomaterials (Basel). 2021 Mar 14;11(3):727. doi: 10.3390/nano11030727 (PMC7998999; doi:10.3390/nano11030727)
Supplement: Supplementary file 1 [file nanomaterials-11-00727-s001.pdf]

Supporting information: Selective oxidation of transient organic radicals in the presence of gold nanoparticles

Viacheslav Shcherbakov, Sergey A. Denisov * and Mehran Mostafavi *

Institute de Chimie Physique (ICP), CNRS/Université Paris-Saclay, Orsay, France; viacheslav.shcherbakov@universite-paris-saclay.fr (V.S.)

* Correspondence: sergey.denisov@universite-paris-saclay.fr (S.A.D), mehran.mostafavi@universite-paris-saclay.fr (M.M.)

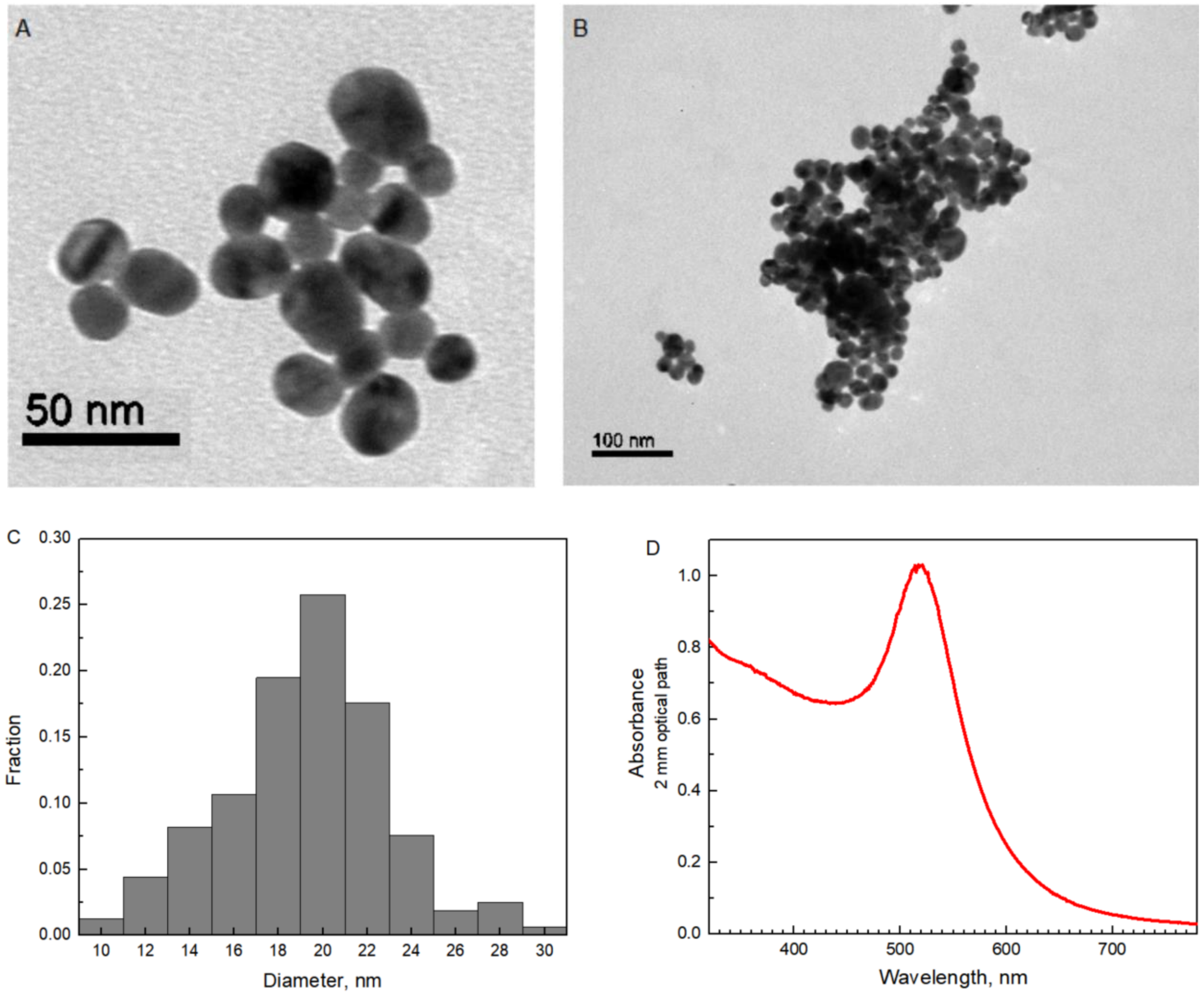

Figure S1. Characterization of AuNPs. TEM image (A and B), size distribution (C) and absorption spectra of AuNPs' suspensions containing 3 mM of gold atoms (D). The suspension was diluted with deionized water two times before measurement.

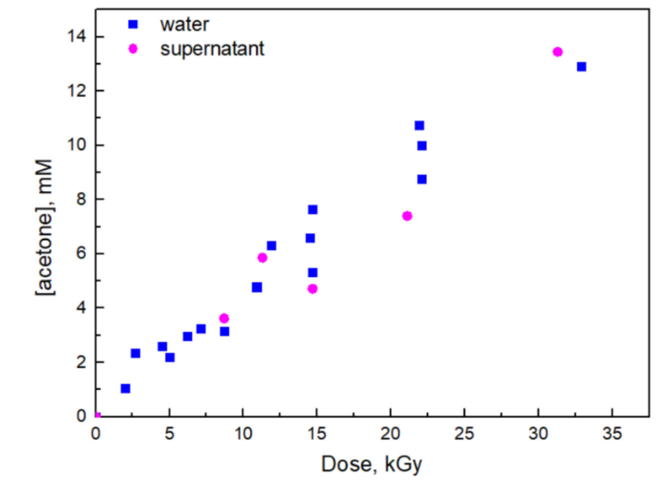

Figure S2. Acetone formation in the aqueous solution of 2-propanol (100 mM) and in a supernatant of AuNPs' suspension containing 100 mM of 2-propanol irradiated by gamma rays at various doses. All samples were saturated with N_2O. The supernatant was prepared by AuNPs precipitation by centrifugation.

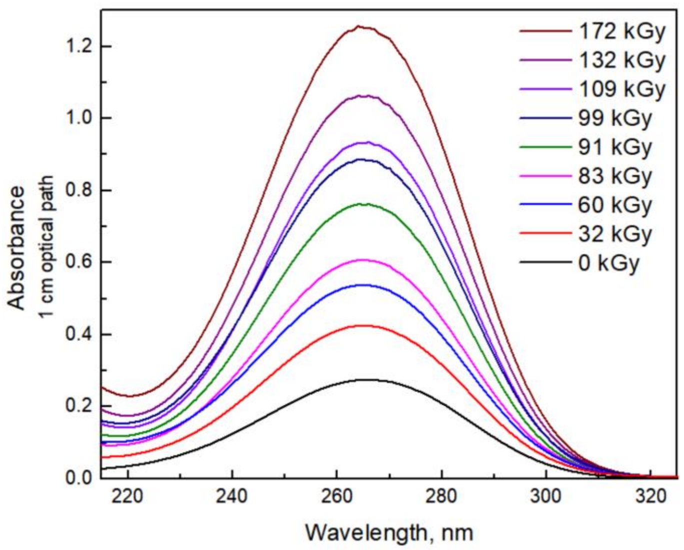

Figure S3. Spectra of AuNPs suspension (12 nM particle concentration) containing 100 mM of 2-propanol and 18 mM of acetone irradiated with gamma rays at various doses. All samples were deoxygenated with Ar.

Reaction S1. Conversion of the hydrated electron (e^-_{aq}) to $^{\bullet}OH$ radical by N_2O.

$$N_2O + e^-_{aq} + H_2O \rightarrow {}^{\bullet}OH + OH^- + N_2$$

Equation S1. The radiolytic yield of $^{\bullet}OH$ radical under N_2O atmosphere.

$$G({}^{\bullet}OH) = G({}^{\bullet}OH) + G(e^-_{aq}) = (2.8 + 2.8) \times 10^{-7} = 5.6 \times 10^{-7} \text{ mol J}^{-1}$$
